# Repeated Gastric Motility Measurement Affects Gastric Motility and Epigastric Symptom Sensation

**DOI:** 10.3389/fmed.2020.00172

**Published:** 2020-04-30

**Authors:** Nick Goelen, John F. Morales, Jan Tack, Pieter Janssen

**Affiliations:** ^1^Translational Research Center for Gastrointestinal Disorders, KU Leuven, Leuven, Belgium; ^2^Department of Electrical Engineering–ESAT, STADIUS Center for Dynamical Systems, Signal Processing and Data Analytics, KU Leuven, Leuven, Belgium

**Keywords:** gastrointestinal motility, gastric balloon, stomach, healthy physiology, medical device

## Abstract

**Background:** Gastric motility is an important determinant of gastric emptying, epigastric symptom generation, and intolerance to food. Motility is classically assessed directly using manometry or an intragastric balloon. These diagnostic methods are perceived as stressful and invasive, which, by itself might influence the readout of these assessments. Our hypothesis was that with repeated exposure to an invasive motility test the outcome would be different.

**Methods:** Gastric motility was assessed with a custom-made orogastric balloon catheter in 10 healthy subjects naive to intubation. A motility index ranging from 0 (no motility) to 1 (maximum motility) was calculated in the fasted state for 3.5 h. Symptoms were surveyed with visual analog scales of 100 mm. Results are presented as median (interquartile range).

**Results:** Motility index during visit 1 [0.40 (0.37–0.59)] was lower compared to visit 2 [0.50 (0.45–0.66); not significant] and 3 [0.63 (0.50–0.71); *p* = 0.016]. Nausea and pain scores were significantly higher during visit 1 (35 (2.8–126) and 103 (88–125) mm, respectively) compared with visit 3 [1 (2.8–26) mm (*p* = 0.016) and 75 (30–100) mm (*p* = 0.008), respectively]. No adverse events were observed.

**Conclusions:** Repeated exposure to an invasive method to assess motility resulted in more vigorous motility and lower symptom scores. Caution is warranted when interpreting functional assessments, as prior exposure to invasive tests might confound the obtained results through habituation.

## Introduction

The human stomach is a powerful muscle. Two very different motor patterns can be distinguished in the stomach: an interdigestive and a postprandial motor pattern. During the interdigestive phase, the proximal stomach muscle tone is high while the distal stomach is engaged in a recurrent phasic contraction pattern known as the migrating myoelectrical (or motor) complex (MMC). This cyclic pattern has three distinctive phases and repeats itself every 90–150 min. The physiological function of the MMC is incompletely elucidated (1).

Upon food intake the motor pattern of the stomach changes drastically: the proximal stomach relaxes and serves initially as a reservoir. This reflex relaxation is referred to as gastric accommodation (2). Consequently, propulsive contractility initially ceases upon food intake. Afterwards, solid particles are grinded and mixed through pro- and retropulsion. Peristalsis ensures gastric outflow of small particles and liquids through the pylorus (3). From a mechanical point of view, gastric emptying of a meal relies on a complex interplay between the major motor patterns of the stomach (4). Various receptors in the duodenum are activated upon delivery of nutrients, resulting in a negative feedback on gastric motility, thereby reducing propulsive contractions and enhancing the pylorus' tone (5).

Assessment of motility might contribute to a more accurate diagnosis of upper gastrointestinal (GI) disorders and guide therapy. A review of the available techniques can be found in the literature (6). Gastric accommodation can be assessed with a barostat device. Orogastric intubation of the barostat bag is burdensome for the patient. Gastric contractility can be assessed invasively with manometry or with an intragastric isometric balloon, connected to a pressure transducer. Gastric contractility results in intra-balloon pressure changes. We recently published the optimal balloon specifications for such assessments (7).

The invasive methods described above are experienced as stressful (8). It is well-established that stress can affect gastric motility (9–12). This is further illustrated by the concept of functional gastrointestinal disorders, which are understood as a biopsychosocial model (13). Repeated exposure to a stressful experience is known to reduce the stress level (14). More specifically for upper GI endoscopy, Essink-Bot et al. demonstrated that repeated exposure can reduce the associated discomfort and psychological burden (8).

As stress and coping, associated with these procedures, might influence the readout of these very procedures, our hypothesis was that in healthy subjects who have never had an oro- or naso-gastric tube (i.e., naive subjects) repeated exposure to an invasive motility test would result in different outcomes.

It was furthermore hypothesized that gastric motility and epigastric symptom burden would change with increasing exposure to the study procedures associated with the isometric measurement of gastric motility.

## Materials and Methods

This was a monocenter, randomized three-way cross-over investigation in healthy adults without prior oro- or nasogastric intubation. Subjects were recruited by means of advertisements in the University of Leuven. Informed consent was provided prior to screening. The absence of chronic dyspeptic symptoms was confirmed using the validated PAGI-SYM questionnaire (15).

**Figure 1:**
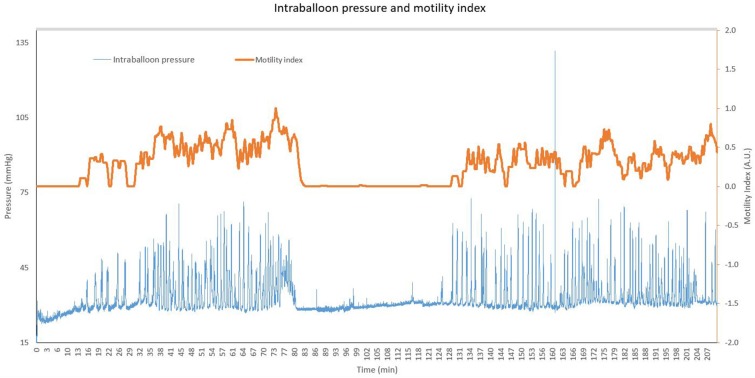
Representative pressure readout and corresponding motility index for a single subject. The original intraballoon pressure is shown in blue (in mmHg, left y-axis) for a period of 210 min. The orange line represents the corresponding motility index, plotted on the right y-axis (motility index ranges from 0 to 1).

After an overnight fast, gastric motility was measured for 3.5 h. Gastric motility was assessed in a continuous fashion using a custom-made orogastric balloon catheter consisting of a standard polyvinylchloride nasogastric catheter and a polyethylene balloon attached to it [KU Leuven, Leuven, Belgium]. Once positioned in the stomach, the balloon was inflated with 180 mL air. The inflated balloon dimensions and intra-balloon pressure are such that intragastric motility can optimally be assessed without inducing epigastric symptoms (7). The single-use balloon catheter was connected to a differential pressure transducer [MPX2050, NXP Freescale, Munich, Germany], a data acquisition unit and software [WinDaq Waveform Browser, DATAQ instruments, Akron, OH, USA]. Custom-made analysis software [Matlab 2017Rb, MathWorks, Natick, MA, USA] was used to filter out artifacts (due to coughing, moving, talking, breathing, etc.) and to identify gastric contractile waves based on a minimum amplitude and a maximum frequency. Gastric motility was quantified based on an automated peak detection algorithm of the contractile waves. A motility index (MI) was calculated as an average of the individual detected contractions in a time window of 2 min, taking into account the relative amplitude of each contractile wave. The resulting MI values were averaged for each subject for the entire recording period (*t* = 0–210 min). The MI represents the fraction of time during which gastric motility was detected, weighed for peak prominence, resulting in a value between 0 (no contraction detected in the respective time window) and 1 (contractile waves occupied 100% of the time window). An example of the original intraballoon pressure readout and respective MI is shown in Figure 1.

Hunger, nausea, bloating and pain were surveyed with 100 mm visual analog scales (0 = absent, 100 mm = worst possible sensation, moderate pain >50 mm) at baseline and at five fixed time-points to assess the safety and tolerance of the investigational device. Baseline scores were recorded prior to intubation. Symptom scores were corrected for the subject's score at baseline. The total score (sum of 5 time points after insertion of the catheter) of each symptom was calculated (ranging from 0 to 500) and compared between three visits. The start time, examination room and investigator were kept constant for all study visits of the same subject.

Results are presented as median (interquartile ranges). Friedman test and Dunn's follow-up test were performed to explore differences over three consecutive study visits. For the secondary endpoint of tolerability, the total symptom burden of each individual symptom was compared between the three visits. A significance level of 0.05 was used.

The study was approved by an Ethics Committee of UZ/KU Leuven (reference S58817). The first subject was enrolled on March 27, 2017. The last visit was completed on August 22, 2018. All subjects gave written informed consent in accordance with the Declaration of Helsinki.

## Results

In total, 11 volunteers were screened and all of them were eligible for participation. One subject could not tolerate the orogastric intubation and withdrew consent during visit one. This dropout was replaced to reach a study population of 10 subjects who have all completed three visits. Due to technical issues, two visits had to be repeated. In total 33 visits were initiated, of which 30 were completed according to protocol. Only data from these 30 visits were suitable for analysis.

Subjects had median age of 22.2 (20.8–23.9) years and median BMI of 22.4 (21.5–24.7) kg/m^2^. The majority were females (7/10).

Concomitant therapies did not result in the exclusion of any of the subjects. Four female subjects used oral contraceptive therapy and one subject used iron supplements as maintenance therapy. No changes were made to the concomitant therapies during study participation

In total, 33 visits were initiated, hence 33 balloon catheters were used. Due to early termination (*n* = 1) and technical issues (*n* = 2), gastric motility was correctly recorded for 3.5 h during 30 visits.

### Gastric Motility

**Figure 2:**
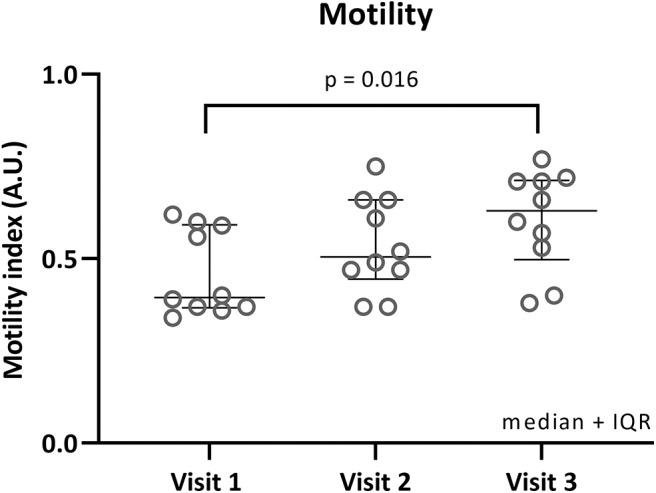
Fasted motility per visit. Individual motility index values in fasted state are plotted for each visit. Median and interquartile ranges are shown as error bars. Significant Dunn's adjusted *p*-values are shown.

Median fasting motility was the lowest during visit 1 [MI: 0.40 (0.37–0.59)], higher during visit 2 [0.50 (0.45–0.66)] and highest during visit 3 [0.63 (0.50–0.71)], see Figure 2. Friedman test showed a significant difference (*p* = 0.012). Follow-up testing (Dunn's multiple comparisons test) showed a significant difference between visit 1 and visit 3 (adjusted *p* = 0.016).

### Safety and Tolerability of Study Procedures

**Figure 3:**
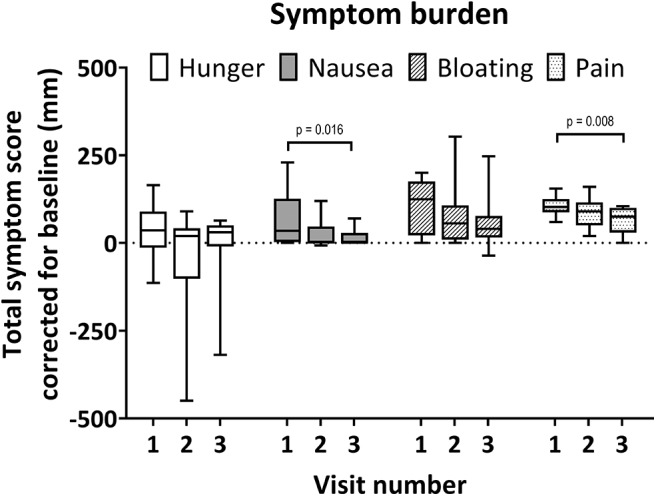
Total individual symptom burden per visit. Boxplot whiskers for minimum and maximum observed values. Scores relative to baseline. Maximal total symptom score relative to baseline is ±500 for each visit. Symptoms were scored on a 100 mm visual analog scale at baseline and 5 time points during each visit. Significant Dunn's adjusted *p*-values are shown.

The total scores (cumulative score of 5 time points) for hunger, nausea, bloating, and pain are plotted in Figure 3. Total hunger scores and bloating scores were not different for the three visits. Total nausea scores were significantly higher during visit 1 compared to visit 3 [median visit 1: 35 mm (2.8–126 mm), visit 3: 1 mm (0–28.3 mm), adjusted *p* = 0.016]. Total pain scores were generally low, however moderate epigastric pain was scored at one instance for a single subject (60 mm, 3.5 h after balloon inflation). Total pain scores were significantly higher during visit 1 compared to visit 3 [median visit 1: 103 mm (88–125 mm), visit 3: 75 mm (30–100 mm), adjusted *p* = 0.008]. One subject could not tolerate the intubation procedure due to retching.

## Discussion

In the present study, gastric motility was assessed with an investigational orogastric balloon catheter in 10 healthy subjects who had never had a prior oro- or nasogastric intubation. The aim was to assess a potential impact of repeated exposure to the study procedure on gastric motility and epigastric symptoms. Furthermore, the safety, tolerability, and feasibility of the study procedures were evaluated. We demonstrated that fasting motility was indeed affected by repeated exposure to the procedure.

Motility was the lowest during the first visit and increased with increasing exposure. The median fasting motility was significantly higher during visit 3 compared to visit 1. No such difference was observed between visit 1 and 2 or between visit 2 and 3, suggesting that a first exposure to the used techniques has an inhibitory effect on gastric motility. Conversely, repeated exposure may stimulate gastric motility in the fasted state. Such an assessment of motility is of special interest in patients with suspected motility disorders, as described in gastroparesis and functional dyspepsia (3). In those patients, the quiescent phase I can be prolonged or phase III can be absent, and both would result in a decreased MI. Currently, no widely available diagnostic technique exists to investigate all aspects of gastric motility (6), thereby hampering the diagnostic and therapeutic management, which are often empirical and symptom based (16, 17). Assessment of motility might contribute to a more accurate diagnosis of upper GI disorders and guide therapy. Newly developed diagnostic procedures might benefit from measuring motility, both in fasted conditions as well as after a nutrient stimulus, since many GI symptoms are associated or aggravated by food intake. The technique applied in the current study has not been clinically validated by lack of golden standard. However, an earlier study by our research group has demonstrated the agreement of the motility readout with simultaneously performed gastric high-resolution manometry to detect phasic contractility (7). Indirect validation of the technique for confirmatory research and clinical purposes is warranted and planned by our research group. Central aspects of validation are to demonstrate the relation between the motility readout and gastric emptying rate and most importantly with clinical parameters. Future research should allow to establish the inter-subject and day-to-day intrasubject variability, both in health and in patients.

This exploratory study was not designed to further investigate the contribution of different factors associated with such a first exposure. We can speculate that psychological (9, 10, 18, 19) and physiological (20) stress contributes to the observed inhibitory effect. Such hypotheses should be tested in confirmatory studies. These initial observations of habituation warrant caution when interpreting data from studies with repeated measures and stresses the value of studies with a cross-over design. The current observations were made in naive healthy volunteers. However, it can be expected that the same factors are present to at least the same extent in (naive) patients as well. It is conceivable that patients are affected to an even greater extent given a potential nocebo effect associated with diagnostic or mechanical investigations (21). The clinical importance is evident as for most patients such an assessment is a first or one-time exposure to oro- or nasogastric intubation. The outcome might also be important to take into account in clinical study design.

Gastric emptying was not assessed in this study. It is well-established that nutrient intake activates a neurohumoral feedback mechanism with extensive effects on gastrointestinal motility to limit the delivery of nutrients to the duodenum (5). Phasic contractile waves associated with the interdigestive fasted state cease (1, 22) while the proximal stomach (fundus and proximal part of the corpus) relaxes to facilitate the intake of a substantial meal volume, without an increase in intragastric pressure, known as gastric accommodation (23, 24).

The use of the orogastric balloon catheter to assess gastric motility did not result in any safety issues. One subject experienced bothersome retching during the intubation and could not tolerate the procedure which resulted in withdrawal of consent after one attempt of intubation. Two visits (both visit number 3) had to be repeated due to a technical software error, resulting in corrupted data files. The exact origin of the technical error was not found. However, the problem was resolved after a reset of the recording software. To allow paired-wise analysis, the two subjects with incomplete datasets repeated one visit each. Hunger and bloating were not different for the three visits. Nausea was significantly more present during visit 1 compared with visit 3. Pain scores were significantly higher during visit 1 compared to visit 3. Importantly, moderate pain (>50 mm) was only reached on a single occasion. One subject experienced mild epigastric pain (60/100 mm). It was concluded that the use of the orogastric balloon catheter was safe and feasible. It should be noted that symptom scores were generally low as a consequence of the included healthy population. The findings cannot be extrapolated toward potential habituation at the level of symptoms in patient populations with chronic dyspeptic symptoms in daily life, and this should be addressed in additional studies.

In this exploratory pilot investigation, factors associated with first exposure, such as psychological or physiological stress, were not assessed. Furthermore, the limited sample size that was arbitrarily defined a priori resulted in a low statistical power and consequently a high probability of Type II errors. Nonetheless, even with this limited sample size and power, statistically significant differences were detected.

These results are highly relevant for clinical diagnosis as well as for research. Invasive motility testing might result in an underestimation of contractile frequency and vigor in naive patients compared to those who have had prior invasive tests or those with a lower stress level. Repeated measures in clinical studies might be affected as well. Our observations stress the importance of crossover placebo-controlled trials.

Based on 31 initiated study visits, it was concluded that the use of the orogastric balloon catheter for the assessment of gastric motility in healthy subjects was safe and feasible. Fasting motility was significantly lower during the first visit compared with the third visit. It was inferred that a combination of factors associated with a first exposure to the study procedures can reduce the frequency and vigor of gastric contractile waves. Caution is warranted when interpreting functional assessments, as prior exposure to oro- or nasogastric intubation might confound the obtained results. The results of this exploratory study prompt further confirmatory research.

## Data Availability Statement

The raw data supporting the conclusions of this article will be made available by the authors, without undue reservation, to any qualified researcher.

## Ethics Statement

The studies involving human participants were reviewed and approved by Ethics Committee Research UZ / KU Leuven. The patients/participants provided their written informed consent to participate in this study.

## Author Contributions

NG, JT, and PJ were involved in the conceptualization of the study, execution of the experiments, and interpretation of the results. JM designed the analysis algorithm and was involved in the processing and interpretation of the results. NG and PJ drafted the manuscript, which was reviewed by all authors. JT was responsible for the overall study conduct.

## Conflict of Interest

The authors declare that the research was conducted in the absence of any commercial or financial relationships that could be construed as a potential conflict of interest.
